# Perovskite Quantum Dot-Based Memory Technologies: Insights from Emerging Trends

**DOI:** 10.3390/nano15110873

**Published:** 2025-06-05

**Authors:** Fateh Ullah, Zina Fredj, Mohamad Sawan

**Affiliations:** Center of Excellence in Biomedical Research on Advanced Integrated-on-Chips Neurotechnologies (CenBRAIN Neurotech), School of Engineering, Westlake University, Hangzhou 310024, China; fatehkhattak@westlake.edu.cn (F.U.); zinafraj@westlake.edu.cn (Z.F.)

**Keywords:** memristor, perovskite quantum dots, halide materials, CMOS process, switching mechanism

## Abstract

Perovskite quantum dots (PVK QDs) are gaining significant attention as potential materials for next-generation memory devices leveraged by their ion dynamics, quantum confinement, optoelectronic synergy, bandgap tunability, and solution-processable fabrication. In this review paper, we explore the fundamental characteristics of organic/inorganic halide PVK QDs and their role in resistive switching memory architectures. We provide an overview of halide PVK QDs synthesis techniques, switching mechanisms, and recent advancements in memristive applications. Special emphasis is placed on the ionic migration and charge trapping phenomena governing resistive switching, along with the prospects of photonic memory devices that leverage the intrinsic photosensitivity of PVK QDs. Despite their advantages, challenges such as stability, scalability, and environmental concerns remain critical hurdles. We conclude this review with insights into potential strategies for enhancing the reliability and commercial viability of PVK QD-based memory technologies.

## 1. Introduction

The exponential developments of cutting-edge technologies, including the Internet of Things, cloud computing, in-memory computing, big data, and artificial intelligence, contribute substantially to data proliferation [1]. Consequently, this imposes exigencies on the computing infrastructure for storing and processing such amplified data [2]. Additionally, the existing computer technology faces fundamental physical challenges owing to Moore’s Law. Firstly, the complementary metal oxide semiconductor (CMOS) technology operates through conventional von Neumann processing architectures where chip memory performance is significantly slower than the processors because of the memory capacity limit and computation separation, resulting in power loss [3,4,5]. Secondly, to achieve increasingly sophisticated process nodes, the integrated circuit industry explicitly aims for nanometer (nm) technology processes [6]. Thus, gates associated with the shorter channels cannot accurately regulate the ON/OFF states of the transistors. Therefore, downscaling can no longer contribute to integrated circuits’ efficiency [7]. In addressing these challenges, cutting-edge computing and digital archives must be established to satisfy the requirements of extremely high computational power and energy conservation.

Memristors, which combine the function of memory and a resistor, represent the fourth fundamental circuit element, and appear robust contenders because of rapid data processing, energy-efficient switching operations, straightforward and CMOS-compatible device architectures, and high-density integration [8,9,10,11]. Most importantly, memristors have demonstrated significant prospects for computing technologies, such as neuromorphic architecture and memory [12,13,14]. Thus, memristors can transcend the von Neumann bottleneck while preserving the principles of downscaling, meeting Moore’s law [15]. Architecturally, they are devices with two terminals whose resistance state can be accurately modulated in response to electrical stimulation. Considering the resistance changes, the memristors are classified as analog or digital circuits [16,17,18]. Since the high resistance state (HRS) and low resistance state (LRS) in digital memristors correspond to “0” and “1”, they are appropriate for rapid processing and data storage. In contrast, analog memristors can constantly process and change electrical signals. Neuromorphic-based networks can also be built through multiplication and accumulation while employing Ohm’s and Kirchhoff’s laws. Mimicking neuronal synapses with memristors via two-terminal devices, the top electrode (TE) symbolizes the pre-synapse, the switching layer signifies the synaptic cleft, and the bottom electrode (BE) corresponds to the post-synapse, respectively [19,20]. Additionally, varying the resistance values can alter the synaptic weight, which governs short-term potentiation and plasticity, respectively, through paired-pulse facilitation and depression. Similarly, Hebbian learning rules, which significantly spike time-dependent plasticity, can also be implemented to regulate long-term potentiation/plasticity, long-term depression, and brain-like functional activities [21,22,23,24]. Since memristive technologies integrate memory with computation, they may find application in artificial intelligence, neural networks, big data and other fields.

**Figure 1:**
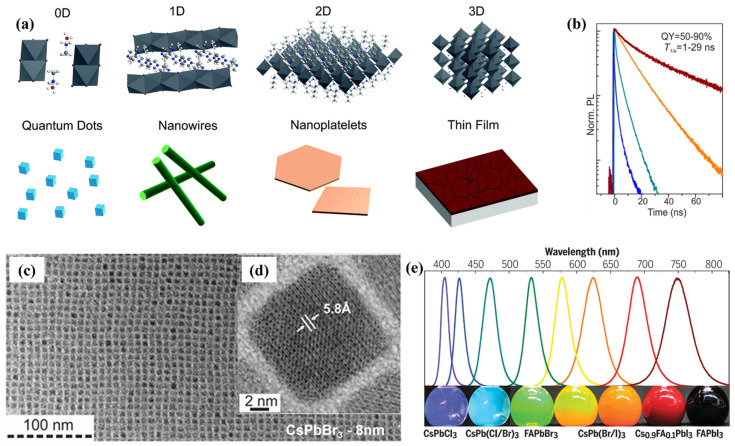
(**a**) Crystal structure and nano morphologies representations [45]; (**b**) the TRPL decay spectra of CsPbX3 QDs (excluding CsPbCl_3_ QDs; (**c**,**d**) the TEM images representing the nanocrystals of CsPbBr_3_ [46]; (**e**) PL spectral survey and mixed UV and daylight image of PVK nanocrystals [47].

Numerous functional materials with resistive characteristics, including metallic oxides, two-dimensional (2D) materials, organics, inorganics, perovskite, etc., have been employed in memristors [25,26,27,28,29]. Although memristors featuring inorganic switching layers are deposited through complex fabrication techniques and significant power consumption, they often display reliable resistance characteristics. Advantageously, organics as a functional layer are versatile and economical and can be processed from their parent solutions; however, they lack adequate durability [5]. In contrast, perovskites (PVKs) demonstrate remarkable characteristics, such as simple yet affordable synthesis and flourishing optical and electrical properties, which are advantageous in building memory architectures [1,30,31]. Three-dimensional (3D) perovskites are generally formulated as ABX3 (Figure 1), where A symbolizes monovalent cationic organics or inorganics, i.e., methylammonium (MA^+^), formamidinium (FA^+^), and inorganic (Cs^+^); B signifies bivalent metallic cations, including Pb^2+^ and Sn^2+^, whereas X are counterbalancing anionic halogens, for instance, I^−^, Br^−^, or Cl^−^, while the overall PVK crystal is electrically neutral [32,33,34]. Thriving due to their outstanding electrooptical characteristics, PVKs provided foundations for diverse memory technologies [35,36,37]. Most importantly, the hysteresis phenomenon in PVKs generated from current-voltage (I–V) characteristics, potentially resulting from migration of ionic–electronic conduction, ferroelectric behavior, or charge traps, encouraged the scientific community to explore PVKs in memory devices [38,39,40]. To date, both unipolar, bipolar and write-once-read-many times memory devices have been fabricated with PVKs as switching layers [41,42,43]. The intrinsic photosensitivity of 3D PVKs has led to photonic memristive devices, in which the device is stimulated by light illumination rather than the conventional electrical bias [44].

Overall, the PVK materials exhibit remarkable capabilities for simultaneously absorbing and emitting photons, and these unique characteristics position them as exceptionally promising candidates for applications that necessitate dual functionality [48]. Additionally, PVKs are crucial in developing innovative and highly integrated optoelectronics developed through the monolithic integration of a photodiode and a light-emitting diode, enabling bias-controlled spectrum-selective photodetection [49]. Additionally, exceptional charge transportation characteristics can be achieved from highly oriented PVK templated over another PVK material through the controlled doping of MAI, which resulted in p-type PVKs, enhancing device performance [50]. In addition, PVK material has the potential to achieve enhanced open-circuit voltages in solar cells, which can be primarily accomplished by interface engineering. More specifically, passivating the interface of PVK/electron transport materials improves the charge extraction and reduces trap densities [51,52]. Moreover, PVKs could be a reasonable choice in achieving a giant dielectric constant for critical electrical components and can be fabricated by the coprecipitation technique [53].

Despite their impressive features, 3D PVKs appeared to have challenges in developing incredibly robust memory architectures. Addressing MAPbI_3_ as photodetectors and resistive switching (RS) memories, the polycrystalline films deposited via solution processing revealed a trap density of 1016 to 1017 cm^−3^, displaying a robust OFF current with a low ON/OFF ratio [54]. Similarly, materialized grain boundaries in polycrystalline films are unstable, especially due to moisture sensitivity, inhibiting reliable, practical device implementation [55,56]. Three-dimensional PVKs with mixed halides encounter phase segregation around grain boundaries associated with photons or electrically energetic ions, resulting in imbalanced and unreliable electrical characteristics throughout the film [57]. Such a plethora of grain boundaries could potentially generate significant defect areas, negatively affecting the electrical characteristics of the devices [58].

Recently, PVK QDs have emerged as an encouraging proposal and have been explored intensively to surmount the outlined challenges. The PVK QDs are crystalline nanomaterials that are easily templated to provide a homogeneous film over a large device fabrication area. Due to their extortionate photonic sensitivity, assigned by their effective surfaces, along with quantum confinement, they are suitable for neuromorphic and photonic applications [59].

This review covers recent developments in memristive architectures employing organic/inorganic halide PVKs QDs, the physical properties of PVKs QDs concerning memory applications, methodologies involved in their synthesis, and mechanistic approaches facilitating a transition between HRS and LRS, along with their respective applications. Conclusions and perspectives are the subject of the last section of this manuscript.

## 2. Memristive Characteristics of Perovskite QDs

Generally, bandgap manipulation in PVKs enhances versatility in their ultimate optoelectronic applications, accomplished either by formulating their chemical entities or fine tuning their nanoscale morphologies [60]. Considering 3D PVKs, bandgap optimizations can be realized either by formulating chemical constituents (manipulating Ionics (A^+^, B^+^ or X^−^ sites) of the crystal structure or regulating their ratios) or by the introduction of the spacer proportions for A^+^–site organics (crystal morphologies are given in Figure 1a) [45]. Correspondingly, in 2D Ruddlesden–Popper PVKs, cationic spacer molecules with long side chains establish quantum wells by separating the octahedral slabs. The dimensionality of 2D Ruddlesden–Popper PVKs can be tailored by regulating the “n” value from 1 to infinity; such adjustment allows the transformation of a purely 2D phase into a hybrid 2D/3D assembly, and ultimately to a more interconnected 3D architecture [61]. Thus, inserting spacing ligands (molecular cations) amends the existing B–X bonds, causing molecular lattices to expand or compress and the bandgap to shift. Similarly, the time-resolved photoluminescence (TRPL) (Figure 1b) can be adjusted by their morphologies and compositions (Figure 1c,d), altering their overall spectral features (Figure 1e) [46,47].

Regarding PVKs, bandgap engineering is fundamental to memory technologies, allowing memory devices to optimize their performance and reliability. Larger bandgap PVKs characteristically exhibit high resistivities, which is advantageous when building memory devices yet detrimental for solar cell applications. Usually, the Schottky barrier is established between the larger bandgap PVKs and corresponding electrodes, often reducing the current in HRS. For instance, Seo et al. fabricated Ag/PVK/Pt memory devices; by engineering dimensionalities from 2D PVK (C_4_H_9_NH3)_2_PbI_4_) to 3D PVK (MAPbI_3_), they observed a decrease in the current (10^−9^ A) at HRS in 2D PVK having a bandgap of 2.43 eV in comparison to the 3D PVK with a bandgap of 1.5 eV and higher current values (10^−5^ A) [62]. This considerably increased the ON/OFF ratio from 10^2^ to 10^7^, and such variation in the current and ON/OFF ratio is caused by thermal activation energy and the Schottky barrier [62]. Additionally, the out-of-plane geometry of the 2D PVKs causes lower conductivities with exceptional ON/OFF ratios (≈10^6^) and reduced sneak currents due to engineering dimensionalities in spatially isolated octahedral slabs. This facilitates memory devices, because charge carriers are limited in a 2D plane [63]. Moreover, memory devices fabricated with halide-bearing PVKs predominantly regulate the mechanism through halide vacancies. Thus, wider bandgaps provide more room and lessen the interaction among the generated halide vacancies [64]. Three-dimensional perovskite encompasses narrower bandgaps; the triggered electronic transition states induced by the halide vacancies will overlap with the conduction bands; thus, the vacancies span the whole conduction band.

Due to their crystalline nature, determined by effective boundaries with minimum defects, PVK QDs reveal superior charge transportation lengths with improved carrier mobilities [65]. Concomitantly, PVK QDs synchronize the fundamental features of 3D PVK and advantageously provide directional quantum confinement, making them an excellent choice for electronic applications. Although ongoing research predominantly involves 3D PVK architectures in search of low-current operative systems, the intrinsic defects associated with grain boundaries are still challenging and typically cause leakage currents [66]. Bandgap increases by engineering 3D PVKs to 2D, 1D and QD due to quantum confinement; therefore, condensation of PVK nanocrystal dimensions beyond the Bohr radius will govern discrete energy states instead of continuous states [65]. Eventually, the quantum confinement effect has the potential to significantly modify the optical feature, giving them inimitable and tailorable optical capabilities [67]. Sichert et al. first investigated the quantum size effect; they regulated the ligand’s content and thickness in exploring MAPbBr_3_ nanoplatelets and observed their corresponding photoluminescence (PL) [68]. Similarly, Imran et al. studied the diameter effects in CsPbBr_3_ 1D nanowires by observing the PL and absorption spectrum [69]. They realized a green-to-blue color transformation while tuning the diameter from 20 nm to 3.4 nm.

The QDs and nanocrystals are crystalline nanostructures; the former has a diameter of about 10 nm, while nanocrystals are a bit larger [70]. In pioneering work, researchers proved that regulating the size of the CsPbX_3_ nanocrystal can direct the bandgap. A shift towards shorter wavelengths (blue shift) indicates a rise in the bandgap energy of the nanocrystals. When the nanocrystal size was reduced from 11.8 to 3.8 nm, a blue shift in both the absorption spectrum and PL edges was observed, increasing the bandgap from 2.4 to 2.7 eV. Studies have revealed that an appropriate crystal diameter stretches the hetero-interfacial exchange region, consequently swinging its absorption edge [71,72]. These key characteristics of PVKs (with low dimensions) are suitable for photonic memory devices, and multi-bit data storage can be accomplished by combining light illumination and electric features [44,73]. Bandgap modification can generally elevate PVKs’ conductivities and coordinating entity densities (intrinsic characteristics), thus, proportional to the memory device performance [74,75]. Notoriously, where coordinating ligands eliminate surface defects and provide stability, long-chain alkylation could probably hinder charge transportation, thus deteriorating the optoelectrical and resistive memory device performance [76,77]. Innovative surface chemistry engineering, such as shorter chain alkylation, could address this concern [78]. In this regard, Bi et al. introduced 2-aminoethanethiol as a partially substituted ligand to CsPbI_3_ QDs, providing a dense ligand extremity around the QDs, preventing film degradation associated with penetration of water molecules and overall receiving higher mobilities with optimal performance [79]. A deeper understanding of PVK QD’s intrinsic characteristics could facilitate resistive device performance.

## 3. Synthetic Approaches

Substantial advancements were made in developing and improving synthetic protocols soon after the first report on PVK QD synthesis [80]. These incredible contributions have implications for refining the consistency and controllability of PVK QDs, which own remarkable ionic features and extraordinary optoelectronic properties [81,82]. This subsequent section provides a concise overview of the synthetic methods involved in PVK QDs. These strategies include ligand-assisted techniques, hot-injection processes, and sol–gel methods, all of which have been fine-tuned to yield PVK QDs with narrow size distributions and high quantum yields [80]. In addition, more advanced techniques, such as microwave-assisted and solvothermal synthesis, have further enhanced the uniformity and stability of PVK QDs, contributing to improved performance in device applications [83]. Another promising approach, the LARP (Ligand-Assisted Reprecipitation) method, has also gained attention for its ability to produce highly uniform PVK QDs with excellent optical properties [84]. Collectively, these advancements have significantly contributed to the refinement of PVK QD synthesis, opening up new possibilities for their application in optoelectronics and other advanced technologies [85].

### 3.1. Hot Injection Method

**Figure 2:**
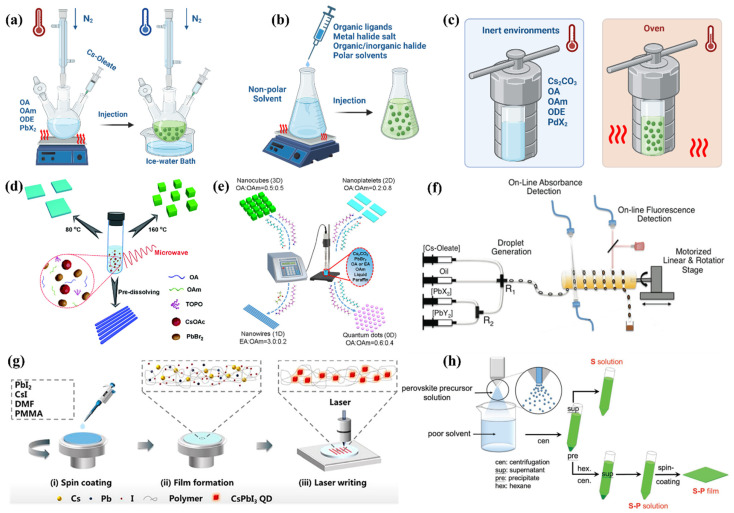
Schematic representation for the PVK QDs synthesis methods: (**a**) hot injection; (**b**) LARP; (**c**) solvothermal; (**d**) microwave-assisted method [89]; (**e**) ultrasonication method [90]; (**f**) microfluidic flow synthesis [91]; (**g**) laser in situ technique [92]; (**h**) spray synthetic procedure [93].

The hot-injection method is a widely used technique for synthesizing PVK QDs, particularly those based on halide perovskite materials, such as CsPbX_3_ (where X = Cl, Br, I) and MAPbBr_3_. This method involves injecting a hot precursor solution into a coordinating solvent, allowing for precise control over the nucleation and growth of quantum dots, which ensures high-quality monodisperse PVK QDs with well-defined sizes and optical properties (shown in Figure 2a). Inspired by traditional semiconductor quantum dot synthesis, this technique enables the fine tuning of material properties, making it ideal for applications in optoelectronics, including light-emitting diodes (LEDs) and solar cells. Building on this approach, Wang et al. synthesized CsPb_1−x_Sn_x_Br_3_ PVK QDs by substituting lead (Pb^2+^) with tin (Sn^4+^) to enhance their stability and photoluminescent properties [86]. This substitution proved essential, as Sn^2+^ is prone to oxidation, leading to instability and low photoluminescence. By carefully controlling the proportion of Sn^4+^, they significantly increased the photoluminescence quantum yield (PLQY) from 45% to 83%, showing that Sn^4+^ doping effectively reduced nonradiative recombination processes and improved the optical properties. The CsPb_0.67_Sn_0.33_Br_3_ quantum dots, synthesized in this manner, emitted light at 517 nm and were incorporated into high-performance light-emitting diodes (QLEDs). These devices demonstrated outstanding performance, with a luminance of 12,500 cd/m^2^, a current efficiency of 11.63 cd/A, and an external quantum efficiency (EQE) of 4.13%, setting a new benchmark for Sn-based perovskite QLEDs. This approach highlights the effectiveness of this synthesis technique in controlling the material properties and enabling the development of stable, high-efficiency devices suitable for real-world applications.

Expanding further on the versatility of this approach, Wu et al. successfully synthesized ultra-small Cs_2_AgBiBr_6_ QDs using a modified hot-injection method and explored their application in the photocatalytic degradation of organic contaminants in ethanol [87]. These Cs_2_AgBiBr_1`_ QDs demonstrated exceptional performance, removing about 97% of Rhodamine B (Rh B) in just 10 min and 60% of tetracycline hydrochloride (TC-HCl) in 30 min under visible light. The degradation efficiency of these pollutants was significantly higher than that of conventional photocatalysts, such as commercial P25 titanium dioxide. Through a series of trapping experiments, the authors identified the main reactive species responsible for the photodegradation: superoxide radicals (·O_2_−) and holes (h^+^). The proposed photocatalytic mechanism revealed that these species played a crucial role in efficiently breaking down ethanol’s organic contaminants, confirming the quantum dots’ superior photocatalytic activity under visible-light irradiation.

Subsequent studies have further advanced the hot-injection technique, particularly in the synthesis of CsPbX_3_ QDs (where X = Cl, Br, I) via a rapid anion hot-injection method. In this process, trimethylhalosilanes are employed as halogen ion donors, enabling precise control over the quantum dots’ composition and emission characteristics. By carefully adjusting the ratios of trimethylchlorosilane, trimethylbromosilane, and trimethyliodosilane, the researchers were able to tune the emission wavelengths across a broad range, from 416 nm to 710 nm, with narrow emission line widths ranging from 13 to 33 nm. Additionally, the luminescence lifetimes of the QDs varied between 32 and 223 ns. Notably, the resulting QDs exhibited enhanced stability in external environments compared to those produced by traditional hot-injection methods [88].

Recently, Zhou et al. successfully synthesized CsPb(Br/I)_3_ PVK QDs using the hot-injection system, which allows meticulous control over the production of high-grade QDs [94]. By incorporating such a process, they successfully achieved a uniform size distribution of the QDs, which is crucial for enhancing the performance and durability of the resulting materials. After synthesizing the pristine quantum dots, they used a dual post-treatment strategy involving 1,4,7,10-tetraazacyclododecane-1,4,7,10-tetraacetic acid hydrate and (2,3-dimercaptosuccinic acid) to address critical challenges related to lead-rich surface defects and halide ion migration, which often affect the efficiency of perovskite devices. Such treatment resulted in a significantly improved photoluminescence quantum yield and stability of the CsPb(Br/I)_3_ QDs. When applied to the production of pure red PeLEDs, the optimized quantum dots exhibited an impressive peak external quantum efficiency of 23.2%, positioning them among the highest-performing mixed halide CsPb(Br/I)_3_-based devices to date.

While the hot-injection method has demonstrated significant efficacy in synthesizing PVK QDs with tunable properties, several limitations exist, particularly when considering their application in memristors. Memristors, which are non-volatile memory devices that rely on resistance switching, require stable and consistent material properties for reliable performance over time. However, the hot-injection technique for synthesizing PVK QDs often presents challenges that could affect their viability in memristor applications [95]. One significant limitation is the scalability of the hot-injection process [96]. Memristor fabrication for commercial applications requires large-area, reproducible deposition of materials. The hot-injection system involves precise control of temperature, precursor concentrations, and solvent choice, which can be difficult to scale up for large-area applications. This could hinder the development of memristors at a commercial level where uniformity and reproducibility across devices are critical. Additionally, the durability of PVK QDs remains a considerable challenge. Memristors require materials with long-term reliability and resistance to environmental degradation, particularly under varying electrical biases and over time [97]. Despite advancements in surface passivation, PVK QDs remain susceptible to moisture, oxygen exposure, and photo-induced degradation, which could compromise their resistive switching properties. To ensure reliable operation in memristor devices, enhanced surface passivation techniques or alternative material formulations are essential to extend the resilience of the synthesized quantum dots. Another challenge is batch-to-batch reproducibility, which directly affects the consistency of resistive switching behavior in memristor applications. Slight variations in synthesis conditions (such as temperature, precursor ratios, and solvent types) can lead to differences in morphological structures and electronic characteristics of PVK QDs, impacting the realization of memristors. For practical applications, achieving consistent, high-quality PVK QDs is essential for the reliable performance of memristor devices.

### 3.2. LARP Method

LARP (Ligand-Assisted Reprecipitation) is recognized as one of the most accurate techniques for synthesizing PVK QDs [98]. This approach involves simply mixing the precursor salts and coordinating diluents and adding them to an antisolvent (depicted in Figure 2b), which induces the precipitation of QDs. The use of ligands helps in regulating the dimensions and structural morphologies of the subsequent QDs, leading to well-defined, high-quality PVK QDs. This technique operates under mild conditions and does not necessitate elevated temperatures or harmful diluents, making it an ecologically intact and economical solution for extensive manufacturing [99]. Expanding on the capabilities of the LARP technique, Ahirwar and Kumar successfully synthesized CsPbBr_3_ perovskite PVK QDs using this method [100]. A precursor solution was formulated with CsBr and PbBr_2_ in dimethylformamide (DMF), which was then mixed with oleic acid (OA) and oleylamine (OLA) as coordinating units. The solution was introduced to toluene (antisolvent), inducing the precipitation of high-quality PVK QDs with controlled dimensions. The resulting QDs exhibited green photoluminescence with a peak at 520 nm and a band gap of 2.36 eV, matching values reported in the literature for CsPbBr_3_. The LARP method, as demonstrated in this work, offers several advantages. It operates at room temperature, which makes it an energy-efficient and simple process for synthesis. The technique is also economical and scalable, making it ideal for substantial production.

Furthermore, it provides excellent morphological control over the shape and dimensions of the QDs, resulting in high-standard PVK QDs with enhanced optical features. In their work, Vázquez et al. applied the LARP to synthesize MAPbBr_3_ PQDs [101]. Briefly, they created a precursor solution of MAPbBr_3_ and ligands, which was then added to toluene as the antisolvent, promoting the precipitation of high-quality quantum dots with precise size and morphology control. The MAPbBr_3_ PVK QDs displayed green photoluminescence at 520 nm and a band gap of 2.36 eV, aligning with typical MAPbBr_3_ properties. In addition, the LARP method offered a cost-efficient and environmentally friendly approach to producing PVK QDs.

Furthermore, Kikuchi et al. focused on synthesizing MAPbI_3_ PVK QDs [102]. They used the LARP method to prepare MAPbI_3_ dispersions, which were purified through a column of molecular sieves to enhance both the optical characteristics and durability of the quantum dots. The passage of the dispersions over the column effectively eliminated acetonitrile and non-luminescent complexes. This purification process significantly improved the photoluminescence quantum yield of the MAPbI_3_ QDs to 88%, with excellent red photoluminescence at 619 nm. Additionally, the photoluminescence quantum yield remained stable at 80% even after 14 days of storage without significant changes in the PL peak or the full width at half maximum. The authors highlighted the simplicity and effectiveness of the molecular sieve purification method, which improved the optical stability of the MAPbI_3_ PVK QDs, making them suitable for optoelectronic applications. This purification method also ensured the size stability of the quantum dots, maintaining their crystallinity and luminescence over time, a significant advantage for real-world applications in displays and lighting technologies.

According to the research conducted by Sanchez et al., the LARP method is a simpler and more scalable alternative to the hot-injection method for synthesizing PVK QDs [99]. While LARP offers advantages in cost-effectiveness and scalability, it is more sensitive to reaction conditions, which can lead to variations in size, morphology, and optical properties. In contrast, the hot-injection technique provides better manipulation over nanocrystal size and optical characteristics, making it preferable for applications requiring high uniformity and performance.

### 3.3. Alternative Synthetic Strategies

Unlike the generally employed HI and LARP procedures for producing PVK QDs, various alternative synthetic strategies have been developed. This diversified toolkit includes solvothermal synthesis (Figure 2c), microwave-assisted synthesis (Figure 2d), ultrasonication (Figure 2e), microfluidic flow (Figure 2f), laser in situ (Figure 2g) and spray synthesis (Figure 2h) methodologies [89,90,91,92,93]. Each process presents its own advantages and challenges, providing opportunities in the design and optimization of PVK QDs. The solvothermal approach involves elevated temperature and pressure in the presence of the solvent to facilitate the nucleation and growth process of PVK QDs [103]. This approach enables superior control in fine tuning the size and uniformity of PVK QDs, as experimental conditions can be finely modified. Furthermore, this process often produces high-quality QDs with a narrow size distribution, making them suitable for various applications.

Another promising method for preparing PVK QDs is ultrasonication, which implements sound waves with high frequencies to induce cavitation in a liquid medium [90,104]. The cavitation phenomenon causes localized high temperatures and pressures in a short time frame, subsequently leading to the rapid formation of PVK QDs. This technique significantly reduces the synthesis time with improved reproducibility and can be expanded (for larger production) for industrial applications. Similarly, microwave-assisted synthesis produces monodispersed PVK QDs by microwave irradiation-assisted heating of the reaction mixtures quickly but uniformly, thus resulting in an efficient nucleation of PVK QDs with uniform and enhanced optical features formation [89,105].

## 4. Fabrication

**Figure 3:**
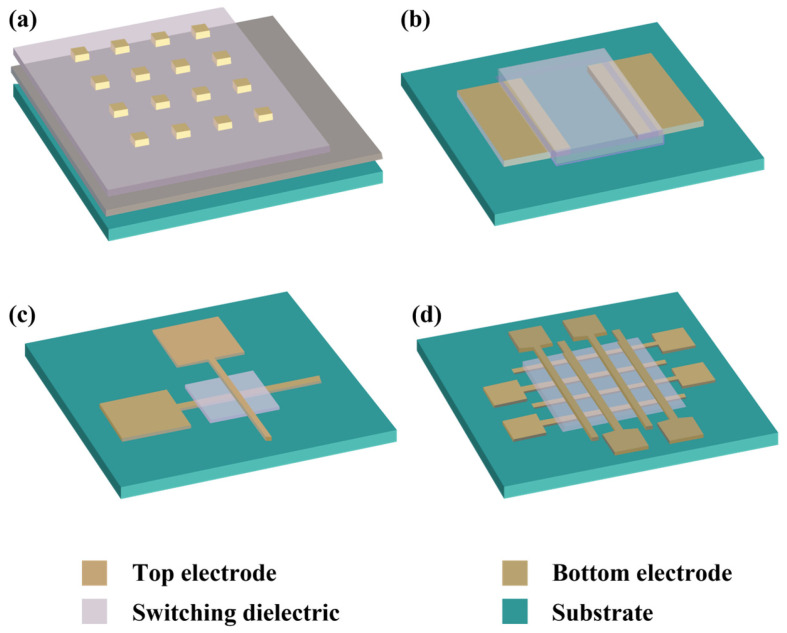
Schematics of memristors: (**a**) single cell configuration, (**b**) lateral configuration, (**c**) cross-point single device, and (**d**) crossbar array architecture.

Memristors have a configuration of metal–insulator–metal, where the dielectric material is confined between the two electrodes. Generally, they are fabricated in an individual capacity (for research objectives) but could be integrated into dense arrays for commercial applications. Figure 3 illustrates the typically implemented architectures.

The PVK QD-based memristors are regularly fabricated as individual cells, sharing a common bottom electrode (BE) (Figure 3a), primarily because of their ease of fabrication and electrical characterization. The fabrication process begins with the deposition of the switching dielectric over the common BE, followed by the deposition of the top electrodes (TE). It is accomplished by precisely placing the shadow mask over the switching dielectric. The metallic probe must be carefully placed on the TE to evaluate the devices, while ensuring the other metallic probe remains in contact with the common BE. The probes should be precisely controlled through micro-positioners. If the precision is not executed, an unintentional penetration of the probe tips could destroy or even move the top TE, potentially leading to a short circuit. As each cell is fundamentally isolated, the architecture is not optimal for integration and can significantly reduce the size scalability. In contrast to vertical device architecture, lateral configurations with horizontal electrodes have also been employed and investigated for mechanistic understanding (Figure 3b) [106]. However, the feasibility of such a configuration is constrained by challenges in attaining a nanoscale channel length. However, some studies achieved a precise channel length in a diagonal direction [107]. It is recommended that performance assessments not be endorsed but rather recommended for fundamental research.

Alternatively, memristors can be fabricated in a cross-point architecture (Figure 3c), where the active cell is positioned at the perpendicular TE and BE intersection. The process begins with a substrate, typically a SiO_2_/Si wafer, which is then coated with a patterned BE, often composed of Au or Pt, using an adhesive layer, such as Ti or Cr, which ensures the adhesion. Following this, the active layer of PVK QDs (or oxide materials) is deposited onto the structured bottom electrode (mostly by spin coating). Finally, the TE is patterned perpendicularly to the BE to complete the cross-point configuration. While this approach demands more intricate fabrication steps, particularly due to the need for precise bottom electrode patterning, it offers advantages in scalability by enabling smaller cell sizes and streamlined testing protocols. This fabrication procedure is complex, as it requires several lithographic steps. However, the reduced footprint and simplified electrical characterization make cross-point architectures promising for high-density memory arrays and neuromorphic circuits. Guidelines often recommend limiting the resistive memory device area to <25 µm^2^ to account for the stochastic nature of conductive filament formation, which predominantly initiates at the most vulnerable sites within the active material [108]. As photolithography, or electron beam lithography, is typically employed, the conventional photolithography faces compatibility challenges with PVK materials due to their susceptibility to degradation. Unlike robust metal oxides, the PVK sensitivity to chemical and environmental stressors has largely restricted the use of lithography in their fabrication, necessitating alternative patterning approaches to preserve material integrity. The crossbar array configuration (Figure 3d) is an expanded cross-point design that integrates multiple parallel TE and BE into a grid-like configuration, enabling interconnected arrays of memory cells. An identical fabrication technique (one employed for cross-point) is used. Still, the process is particularly challenging for PVKs due to their vulnerability to polar solvents. Despite these hurdles, crossbar arrays are prioritized for commercialization, as they better emulate real performance when integrated. A critical limitation, however, is the sneak current issue, where unintended current leakage through adjacent cells disrupts the target cell’s operation. To overcome such interference, advanced integration schemes, such as 1T1R (one transistor + one resistor), 1S1R (one switch + one resistor), and 1D1R (one diode + one resistor) configurations, have been proposed to isolate cells and enhance operational reliability [109] electrically. These strategies aim to address key challenges in scaling memristive technologies for high-density memory applications.

## 5. Switching Mechanisms

Fundamentally elucidating switching mechanisms (between HRS and LRS) in PVKs is cardinal for device design strategies and optimal performance. Several switching mechanisms have been suggested and employed in elucidating the memory phenomenon in PVKs; often acceptable approaches are either ionic migration [either cationic (Ag^+^) or anionic (iodide ions I^−^)], charge trapping and de-trapping. This section will elaborate on these prevailing mechanistic approaches regarding PVKs.

### 5.1. Ionic Migration Mechanism

**Figure 4:**
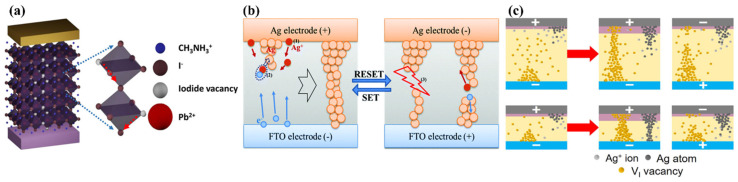
(**a**) Schematic demonstrating VCM mechanism by illustrating the iodide vacancies migration across the periphery of the perovskite octahedra [113]; (**b**) the ECM illustration exemplifying Ag filament formation and dissolution [118]; (**c**) double-filament model featuring Ag/MAPbI_3_/FTO device having a thicker (upper) and thinner (lower) MAPbI_3_ layers, showcasing the synchronicity of VCM and ECM within a single RS architecture [119].

Generally, in PVK-based solar cell devices, the hysteresis obtained from current-voltage (I–V) characteristics directs the mechanism [110]. For instance, research revealed that under external bias, both I^−^ and MA^+^ ions are drifting in MAPbI_3_-based solar cell devices [111]. For PVK resistive switching (RS) devices, mechanistically, the conductive channel formation occurs either by electrochemical metallization mechanism (ECM) or valence change mechanism (VCM). The transitions between HRS and LRS via VCM (sandwiching PVK between two inert electrodes) are triggered by either halide vacancies or associated interstitials [112]. The charged halide ions drift through grain boundaries towards the bottom electrode by applying an external positive voltage to the RS architecture’s top electrode. Thus, the relocation of halide ions will generate halide vacancies; with the accumulation of such vacancies at the interface, a conductive channel or filament is formed, transitioning the device from HRS to LRS. Subsequently, passing an opposite voltage will cause the halide vacancies to recombine with the halide ions, thus breaking the formed filament (reverting the device back from LRS to HRS). This mechanistic model was developed by Gu et al., revealing that iodide vacancies can be moved across the edge (presented in Figure 4a) of the octahedra (PVK crystal); thus, their accumulation governs a filament [113]. The literature revealed that the transition between HRS and LRS (PVKs through VCM) could initiate a smaller external bias owing to the small activation energies (EA) associated with halide vacancies [110].

Theoretical analysis also showcased a fascinatingly low EA (0.08 eV) for iodide vacancies, which ensured the establishment of the conductive channel while requiring the least operating voltage [114]. While many studies attribute resistive switching in perovskites to either VCM or ECM, several reports indicate overlapping behaviors, especially in devices using Ag electrodes, where both halide ion migration and metal filament formation may coexist. This raises questions about the dominant mechanism in hybrid architectures and suggests a need for in situ validation methods.

Lu et al. directly observed filament development by energy-dispersive X-ray spectroscopy (EDX) [114]. The EDX analysis characterized the uniform iodide chemistry in the HRS state, while a gradual decline was observed in the EDX signal in LRS, indicating that the filament formed by iodide vacancies. Recent research conducted by Gonzales et al. explained the spectral properties of PVK (2D Ruddlesden–Popper) memristors [115]. They found that transition-switching dynamics were attained through spectroscopic analysis via impedance spectroscopy. Chronoamperometric measurements monitored gradual transitions for Ag-based RRAM, yet revealed an abrupt switch for Spiro/Ag-based RRAM. The variation in the kinetic phenomenon was attributed to the ionic movement and relocation occurring at the boundary between the PVK and the metallic contacts. In their follow-up study, they studied the mechanistic and kinetic analysis of the fabricated devices with buffer layers (6,6-phenyl C61 butyric acid methyl ester, PCBM, or PMMA) between the PVK matrix and the Ag electrode [116]. It was observed that there were instances of both sudden and gradual changes in behavior. The charge accumulation and reaction happening at the interface govern both drift and diffusive switching behavior. A gradual upsurge in the current was observed, which is associated with halide migration through the PVK matrix, while an abrupt transition is linked to the Ag^+^ filament formation. Lee et al. also observed that the device switching by VCM in QD-based devices transformed from *α*-CsPbI_3_ [117]. This could be attributed to the substantial number of iodine vacancies, facilitated by the high surface-to-volume ratio, hence allowing iodine vacancies to migrate with ease and resulting in the formation of VCM.

In the ECM mechanism, reduction and oxidation of the electrochemically dynamic metals, including Ag, Cu and Ni, contribute crucially to establishing and breaking the conductive filament. The active metals are generally deposited as top electrodes, while noble metals containing Au and Pt are placed as bottom electrodes. By supplying an external positive bias through the upper active electrode, the metallic entity undergoes a process of oxidation into cations, and the electric field influence will cause this cationic migration to the material layer. The metallic cations are then reduced to the respective metals upon embracing the bottom electrode.

A gradual accumulation of these metal atoms forms a conductive channel, thus causing a shift from HRS to LRS. However, when the bias polarity is reversed, it oxidizes the metal atoms adjacent to the upper dynamic electrodes, and the dissolution of the conductive channel will be accomplished by transitioning the device back to HRS from LRS. Yan et al. experimentally observed that memristive features require electrochemically active metals [120]. They also demonstrated that resistance at LRS is temperature-dependent, thus confirming the active metal participation in the device’s nature.

In another study, Yoo et al. also drew similar conclusions through the electrical characterization of Ag/CH_3_NH_3_PbI_3−x_Cl_x_/FTO (fluorine-doped tin oxide) memristive architecture (Figure 4b) [118]. They demonstrated a linear response between LRS resistance and temperature, thus verifying Ag accountability in resistive behavior. Sun et al. provided thickness-dependent resistive analysis in Ag/CH_3_NH_3_PbI_3_/Pt, revealing competition among the Ag conductive channels and iodide vacancies (Figure 4c) [119]. As the film thickness increases, the Ag migration decreases, and only iodide ions and vacancies are involved in the conductive channel formation, which could be attributed to Ag’s lower electric field requirement. In 2020, Yao et al. directly visualized the conductive channels [121]. They observed the expansion of Ag metal dendritic microstructures in Ag/PMMA@CsPbI_3_/Ag utilizing a scanning electron microscope. A concise table summarizing the key features of ECM, VCM, and interfacial switching (see Table 1).

### 5.2. Interface-Type Mechanism

**Figure 5:**
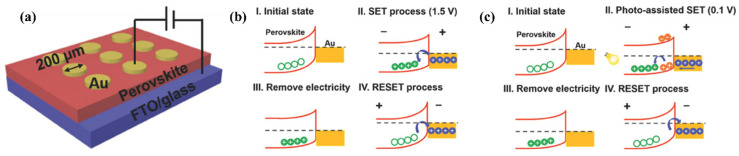
(**a**) Perovskite-based random-access memory schematics. (**b**) Electrical switching mechanism. (**c**) A photo-assisted mechanistic approach is detailed, featuring four distinct states: (I) the initial state representing HRS: characterized by the presence of hole capturing reservoirs situated at the surface of the perovskite material; (II) the SET process: where corresponding traps become filled, shifting the Fermi level near to the valence band; (III) elimination of the light electricity: leading to the formation of a reduced barrier and quasi-ohmic (the LRS); and (IV) resetting process (electrically): extracting holes as of their trapping reservoirs, transitioning back to HRS [122].

In an interfacial-type mechanism, the transition between the resistive states initiates between the switching dielectric and electrode material (at the energy barrier) [124]. Characteristically, a Schottky barrier prevails at the boundary of the metallic electrodes and the semiconducting materials, which can be effectively modulated by tuning the electron affinities of the semiconducting materials and the electrode’s work functions [125]. The Schottky barrier and the contact’s resistance could be altered by causing Fermi-level pinning through metal-induced gap states [126]. Eventually, a homogeneous resistance over the entire electrode region can be achieved. The differentiation between filamentary (independent of the cell area) and interfacial types (having an inverse relation with electrode area) could be observed by measuring an area-dependent resistance. Guan et al. employed MAPbBr_3_ as PVK material in an interface-type memory device; device analysis suggested that methyl ammonium (MA) migration modulates the Schottky junction at the interface of ITO/MAPbBr_3_ [30]. Zhou et al. introduced mixed halide perovskite (MAPbI_3−x_Cl_x_) positioned between the Au and FTO electrodes (shown in Figure 5a) [122]. Upon increasing the external bias, Au-injected holes are filled at the Au/MAPbI_3−x_Cl_x_ interface traps, eventually lowering the Schottky barrier. The established interfacial contact turns into quasi-ohmic, switching the device into LRS. The deep-level defects could retain the injected holes even if disconnected from the external voltage. With a negative voltage, the holes can be recovered from corresponding traps, transmuting the Schottky barrier to elevate its level and returning the device to the HRS. In another work, Han et al. fabricated all-inorganic (CsSnI_3_) memristive devices [123]. They introduced Ag and Au top electrodes, where the former revealed filamentary switching through Ag filament formation. Similarly, the Au/CsSnI_3_ interface gradually changed its resistance due to the interfacial mechanism attributed to Sn vacancies’ influence on lowering the Schottky barrier.

## 6. Memory Applications

Throughout the past twenty years, memory speed has improved by nearly 10% yearly, while processor speed has improved by approximately 55% annually [127]. This has led to a significant imbalance in memory development, which is now developing slower than the processor’s computational speed. This restriction brought on the cost of memory performance known as the “Memory Wall [128,129]. Accordingly, novel device architectures have been employed, such as resistive random-access memory (RRAM), phase-change memory, and ferroelectric random-access memory [130,131,132,133,134]. RRAM could be the best choice in advancing memory technologies due to its simple device architecture, ease of fabrication, and excellent data retention with nanosecond operations [135]. Although many devices boast high ON/OFF ratios and long retention times, their performance can vary significantly depending on the synthesis method, quantum dot (QD) size, and device architecture. For example, MAPbBr_3_-based devices demonstrate excellent retention yet exhibit cycle-to-cycle variability, particularly under ambient conditions. These inconsistencies underscore the necessity of unified benchmarking practices and a deeper understanding of interface effects.

Compared to 3D bulk counter material, PVK QDs in nanometer dimensions exhibit exceptional features, such as bandgap, absorption, and emission tuning abilities originating from quantum confinement [136]. Employing PVK QDs as a medium for ion transportation and information storage in RRAM, the transition between HRS and LRS (referring to 0 and 1 in the logic terminology) could be achieved by external bias.

**Figure 6:**
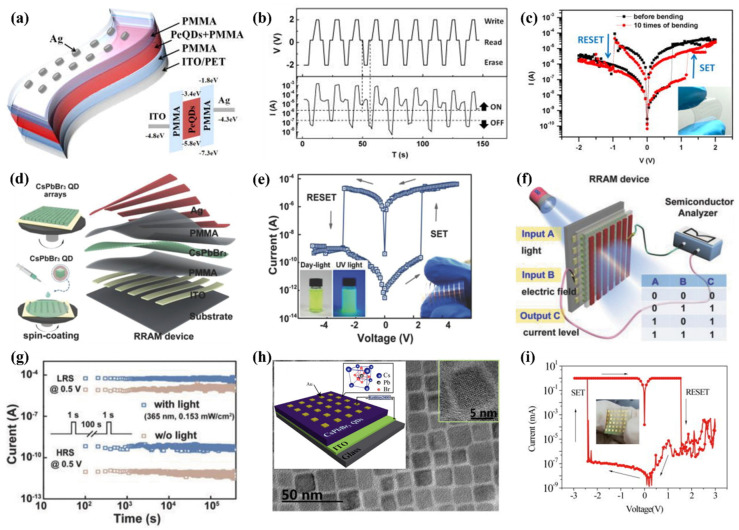
(**a**) Diagrammatic representation of PVK QDs flexible memory architecture, inset: energy level presentation; (**b**) devices’ results showing erase–read–write–read; (**c**) I–V curve attained before and after 10th bending, inset: photographic illustration in bending state [137]; (**d**) graphics of the CsPbBr_3_ QD-based RRAM, fabricated via solution processing; (**e**) the I–V characterization: inset (right) device photograph and (left) image showing PVK QDs solution under daylight and UV light illumination, respectively (**f**) pictorial representation of the CsPbBr_3_ QD-based logic OR device (**g**) retention characteristics with and without light exposure [139]; (**h**) the TEM image of CsPbBr_3_ QDs, inset: HRTEM and device structure; (**i**) RS behavior of the device, inset: corresponding image of the memory device [140].

Recent advancements in the field of optoelectronics employed perovskite materials, especially organic–inorganic hybrid PVK composites [136]. In 2017, Yang et al. developed a flexible polyethylene terephthalate (PET)/ITO substrate and a hybrid memristive device with MAPbBr_3_ PVK QDs (Figure 6a–c, data provided in Table 2) [137]. To prevent the aggregation of the individual QDs, a nanocomposite of MAPbBr_3_ QDs and polymethyl methacrylate, PMMA) was produced. The MAPbBr_3_ PVK QDs-based device displayed an outstanding electrical characteristic, revealing an ON/OFF ratio of 10^3^, and a retention of around 4000 s. Attributed to the virtuous dielectric and insulating hallmarks of PMM operating at a speed of 10 ns A, it is a ubiquitous matrix substance for building memristors [137]. An et al. explored a memristive device with PMMA embedded with all-inorganic PVK QDs, using aluminum (Al) as the top electrode (Al/CsPbCl_3_ QDs: PMMA polymer/ITO) [138]. The electrical measurements (I–V curves) provided an ON/OFF value of 2 × 10^4^, whereas the retention was 10^4^ s. Furthermore, the optimized PMMA matrix elucidates the operational mechanism; the carrier transportation adhered to the SCLC mechanistic approach at HRS, and LRS followed the Ohmic conduction at the cost of filament formed by bromide (Br^−^) vacancies.

Considering photonic RRAM, Wang et al. also fabricated a memristive device with PMMA sandwiching the CsPbBr_3_ QDs in the architecture PET/ITO/PMMA/CsPbBr_3_ QDs/PMMA/Ag [139]. It was demonstrated that a pronounced RS behavior could be realized from the generation and eradication of metallic conductive channels and Br^−^ vacancies when subjected to external bias and light illumination. In addition to the concentration of CsPbBr_3_ QDs, applied external bias and compliance current (CC) can alter the switching voltages (Figure 6d–g). The switching mechanism was investigated by field emission scanning electron microscopy (FE-SEM) and energy-dispersive X-ray spectroscopy (EDX) characterizations, suggesting that electrochemical metallization and valence change phenomena are involved. The devices fabricated with flexible substrates demonstrated an elevated ON/OFF ratio of 6×10^5^. Furthermore, linking the corresponding PVK QDs memory device with a p-channel transistor functions as a flash memory analogue. Subsequently, Chen et al. also employed CsPbBr_3_ QDs as a switching material in a simple sandwiched memristive device (Au/CsPbBr_3_ QDs/ITO) via solution processing (Figure 6h,i) [140]. The device demonstrated remarkable repeatability with a vast memory window by achieving data retention of around 1000 s and approaching an ON/OFF ratio of 10^7^. The HRS of the memristive device can be adjusted through light illumination, and a very low reading voltage (−0.3 V) is required to switch on the device. Finally, it was determined that the Br^−^ vacancies, at the expense of electric fields, were accountable for the transition course (making and breaking of the conductive filament).

**Figure 7:**
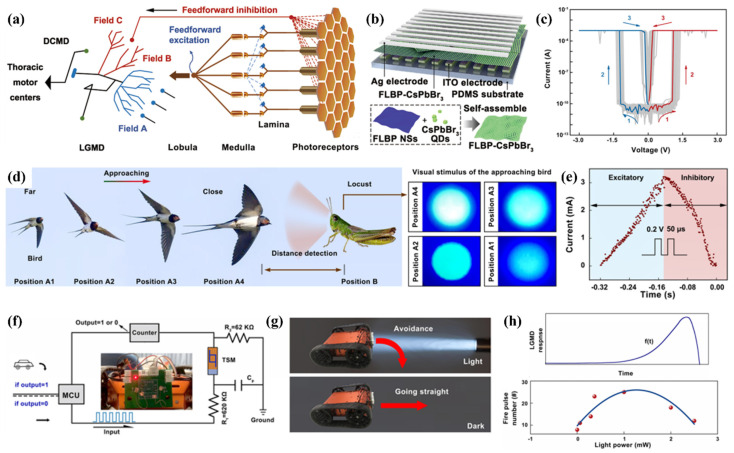
(**a**) Schematic representation of the anatomy of the vision system featuring the LGMD, showing the photoreceptors arranged in packed hexagons within the locust’s compound eye, capturing light stimuli and delivering the visual signal through an electrical impulse through retinotropic layers comprised of lamina/medulla/lobula. Field A of LGMD correspond to receiving the feedforward excitation, and fields B and C correspond to the feedforward inhibition; (**b**) diagrammatic illustration of the TSM; (**c**) a unipolar TS behavior of 100 TSM cells; (**d**) illustration of a bird approach in the direction of the locust and images of the visual stimuli perceived by the locust, illustrating a steady rise in optical intensity from position A1 to A4; (**e**) the excitatory and the inhibitory responses received from the device to a looming light stimulus while concurrently receive the programmed electronic pulses (characterized at 0.2 V, a duration of 50 μs with and interval of 50 μs; (**f**) the schematic of a model car testing configuration, inset: provide a rear view of TSM on a printed circuit board; (**g**) illustration of decision making for the robot vehicle equipped with optical signal processability; (**h**) the upper part demonstrates the time evolution of f(t) functioned LGMD response, while the lower part statistically reveals the firing pulses at variable power supplies, ranging from 0.00 to 2.5 mW [59].

Lobula giant movement detector (LGMD)-modelled vision chips are typically developed and executed in very large-scale integration (VLSI) frameworks, incorporating slabs of retinotopic components, static random-access memory (SRAM) units, and field-programmable gate array (FPGA) dais. Recently, the function of LGMD neurons has been replicated by stacking a single device with molybdenum disulfide (MoS_2_) as a photodetector over floating-gate memory [141]. However, mimicking LGMD neurons with substantially large, energy-consuming, and complex transistor-based processors integrated into a single device is challenging. Therefore, for simulating LGMD neurons, developing devices featuring a relatively compact design and straightforward operational mode is cardinal. Drawing inspiration from visual neurons of LGMD, Han’s research group documented light-modulated threshold memristors (TSMs) to develop biomimetic compound eyes [59]. The TSM arrays consist of a singular device architecture featuring a heterostructure of Ag (top electrode) on the top of a few-layer black phosphorus nanosheets and CsPbBr_3_ PVK QDs, integrated with ITO (bottom electrode) (Figure 7a–h). LGMD, a motion-sensitive neuron located in the third visual neuropile of the lobule, can detect approaching objects and swiftly initiate getaway responses. They fabricated a singular two-terminal artificial neuron with a few-layer black phosphorus and CsPbBr_3_ PVK QDs TSM. A device of this nature demonstrated an exceptionally high ON/OFF ratio exceeding 10^7^, accompanied by the standard I–V characteristics. The impulses to the light flow field, encompassing excitatory and inhibitory reactions, were triggered by the dynamic process of making and breaking conductive filaments associated with Ag metal. These conductive filaments demonstrate a crucial response in the neural response mechanism, drawing an analogous response observed in LGMD neurons. In addition to the neural responses, the biomimetic compound eye exhibited impressive capabilities with detection capability over an expanded view of 180° in both horizontal and vertical dimensions. Such an extensive field of view is principally advantageous for detecting approaching objects and, thus, enhancing the response to identifying potential threats from various angles.

**Figure 8:**
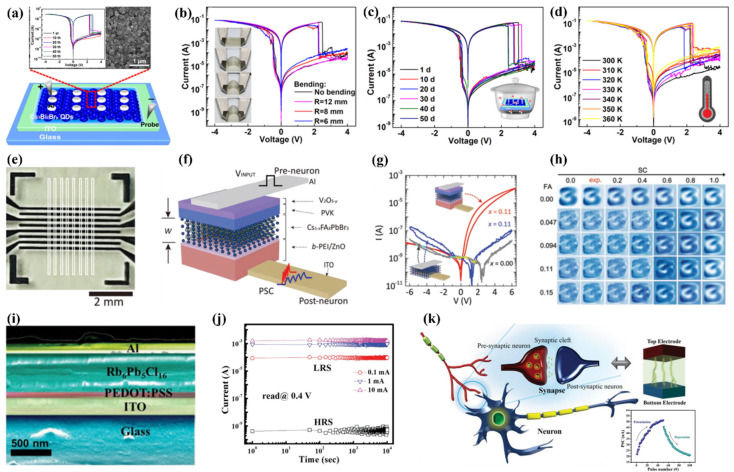
(**a**) Diagrammatic representation of the memristive device and its corresponding top-view SEM image, inset: I–V curve for 50 cycles; (**b**) I–V behavior of the flexible device; (**c**) I–V characteristic of the device unsealed at 60% humidity and as a function of storage time; (**d**) I–V characterization conducted at various temperatures under normal conditions [142]; (**e**) optical photograph of the crossbar array architecture; (**f**) schematics of the device showing the materials involved; (**g**) I–V plots for Al/Cs_1−x_FA_x_PbBr_3_ QD/ITO (x = 0.00 and 0.11) and Al/V_2_O_5–y_/PVK/Cs_1−x_FA_x_PbBr_3_ QD/b-PEI/ZnO NCs/ITO (x = 0.11); (**h**) rewritten 28 × 28 contour images corresponding to the digit “3” concerning the number of learning epochs as a function of FA concentration (0.00 ≤ x ≤ 0.15) [152]; (**i**) cross-sectional FESCM; (**j**) retention observed at various states of the device; (**k**) schematics of the signal transmission process in biological neurons and LTP and LTD emulation [153].

Considering environmental concerns, researchers strive to replace lead components in inorganic PVK QDs for memory architectures. Currently, very little research is focused on lead-free PVK QDs, as memory technologies based on inorganic PVK QDs are still in the early stages of development. Therefore, transforming PVK QDs into lead-free, safe, and reliable memory technologies is becoming increasingly attractive. However, their substandard memory performance compared to their lead-based counterparts hindered their commercial viability. Cao et al. recently developed a lead-free memory device featuring Al/Cs_3_Bi_2_Br_9_ QDs/ITO architecture (Figure 8a–d) [142]. The Cs_3_Bi_2_Br_9_ QDs were created by modifying the ligand-assisted recrystallization method. Incorporating Cs_3_Bi_2_Br_9_ QDs into RRAM devices made it the first of its kind. A form-free, nonvolatile behavior was observed, yielding a high ON/OFF ratio of approximately 10^5^. The transition from HRS to LRS began by sweeping from a negative voltage of approximately −0.45 V to about 2.2 V, demonstrating a bipolar resistive character. Simultaneously, flexible, reliable, and reproducible devices that withstood moisture, heat, and light were developed and operated under these conditions for up to 200 days. Remarkably, the devices exhibited resistive properties when exposed to light, enabling the execution of logic gate functions, such as “AND” and “OR”, suggesting their potential in logic systems that integrate both storage and processing capabilities.

Binary oxides, particularly graphene oxide (GO), have been widely used to construct flexible and transparent RRAM due to their attractive features, such as flexibility, photosensitivity, thermal resistivity, and dielectric properties [143,144,145]. However, the reported ratio between the HRS and LRS is below 10^4^, which limits their application in low-power storage with high capacities for big data processing [146,147]. In contrast, PVKs exhibit flexibility and exceptional photoelectronic characteristics, making them promising candidates for photoelectrically controlled RRAM [122,140,148]. Zhao et al. presented a flexible and transparent RRAM device by employing CsPbBr_3_ QDs along with GO (Figure 6) [149]. Upon illumination, the RRAM device (Ag/CsPbBr_3_ QDs: GO/ITO) demonstrated a high ON/OFF ratio of approximately 1.4 × 10^7^. The resistivity of the flexible device slightly decreased due to the bending cycles. Even after 10^4^ bending cycles, the ON/OFF ratios remained consistent, recorded at 2.5 × 10^7^ and 2.3 × 10^7^, respectively.

Mixed-dimensional heterostructures (integrating reduced dimensionalities, such as 0D and 3D) could significantly advance reconfigurable switches and neuromorphic functions [150,151]. It is anticipated that such heterojunctions could cause abrupt variations in energy dependence on the density of states and the degree of electrostatic screening at mixed-dimensional heterointerfaces and could substantially modulate their electro-optical, magnetic, and thermal properties, thus offering desirable features for imminent technologies. From this perspective, scheming heterostructures with mixed inorganic PVKs with low dimensions could be a straightforward approach to designing selector-free inorganic PVK memristive arrays. Furthermore, regulating the dopant and stoichiometric ratio during bottom-up synthesis may enhance the electrical characteristics and improve learning capabilities, specifically towards recognition accuracy and learning speed. However, investigations concerning PVK heterostructures with mixed dimensions employed in memristors are scarce; thus, their electro-optical behavior is not well understood. Therefore, stoichiometric and doping effects on learning capabilities are rare. Park et al. implemented synaptic arrays employing mixed dimensions of PVKs via dopant amount in selector-free analog RS characteristics with enhanced learning ability [152]. The memristive crossbar array (8 × 8) applied Cs_1−x_FA_x_PbBr_3_ QD as a switching matrix, and the doped material concentration varied between 0.00 ≤ x ≤ 0.15 for FA = formamidinium cation. The resulting individual cells (64/64) of the array performed a self-rectified and electroluminescent behavior, which was absent in the Cs_1−x_FA_x_PbBr_3_ QD-only junction (Figure 8e–h). This self-rectified and light-emitting behavior was achieved by the establishment of an asymmetric barrier at the interface, either at the electron transport layer (ETL) or hole transport layer (HTL) of the mixed-dimensional PVK QDs, and the degree of charge carrier transportation (e^−^, h^+^, FA^+^, and FA vacancies) responding to the polarity as well as the amplitude of the provided bias. Additionally, by increasing the x-value within 0–0.11, the recognition performance of the Modified National Institute of Standards and Technology (MNIST) reached 89.08% from 68.97%. Moreover, the essential power consumption in attaining a specific level of accuracy diminished by a factor of 25.15 while significantly reducing the learning periods (from 256 to 6).

From a material perspective, instead of cesium (Cs), an A-site cation in the ABX3 PVK formulation, rubidium (Rb) has also been introduced for various electro-optical dimensions [154,155]. However, due to Rb’s small ionic radii, the resulting PVK lattice might become distorted, leading to different PVK or non-PVK phases [156]. Interestingly, RbPbI_3_ is a non-switching orthorhombic PVK at room temperature (lacking intrinsic vacancies), while pure RbPbBr_3_ and RbPbCl_3_ PVK states have not yet been documented [157,158]. Although nanometric Rb_6_Pb_5_Cl_16_ has been reported and smaller Cl^−^ ionic radii could be advantageous in inducing trap states, it has never been used as a switching layer in memristors [153]. Das et al. presented Rb_6_Pb_5_Cl_16_ as a switching matrix in memristive devices [159]. Their fabricated architecture involved rubidium-based PVK QDs (Rb_6_Pb_5_Cl_16_) as a functional layer. The results attained from the Al/Rb_6_Pb_5_Cl_16_ QDs/ITO device clarified that the SET voltage fluctuated but stabilized after a few cycles (described in Figure 8i–k). Surprisingly, the device failed to maintain a stabilized state after the SET voltage was applied, shortly after the measurements were taken, within a few minutes. This inconsistency could be attributed to the self-rejuvenation of the pristine device state, thus allowing relaxation time for ion redistribution within the matrix. To prevent self-recovery, a buffer layer (poly(3,4-ethylene-dioxythiophene) polystyrene sulfonate, PEDOT:PSS) was incorporated. The buffer layer functions as a charge reservoir, where migrated ions can be permanently restrained. Recently, Lee et al. synthesized *α*-CsPbI_3_ QDs and applied them to memory devices utilizing an inert Au electrode [117]. The RRAM devices demonstrated reliable I–V curves with 800 switching cycles and retention exceeding 4 × 10^4^ s.

**Table 2:** Summary of the PVK QDs application in memristive research.

Device Structure	Organic/ Inorganic	Vset/VreSet (V)	Ion/Ioff Ratio	Endurance (Cycles)	Retention (s)	Mechanism (Proposed)	Flexible (Bending Cycles)	Ref.
ITO/PMMA/CH_3_NH_3_PbBr_3_/PMMA/PMMA/Ag	Organic	1/−1	10^3^	–	4 × 10^3^	Trap-assisted SCLC	10	[137]
ITO/CsPbCl_3_: PMMA/Al	Inorganic	−0.3/2.6	2 × 10^4^	100	10^4^	Trap-assisted SCLC	No	[138]
PET/ITO/PMMA/CsPbBr_3_/PMMA/Ag	Inorganic	2.6/−2.7	6 × 10^5^	5 × 10^3^	4 × 10^5^	ECM and VCM	100	[139]
ITO/CsPbBr_3_/Au	Inorganic	−2.4/1.55	10^7^	–	10^3^	VCM	No	[140]
PDMS/ITO/FLBP-CsPbBr_3_/Ag	Inorganic	1.05/0.2	10^7^	–	–	ECM	–	[59]
PET/ITO/CsPbBr_3_: GO/Ag	Inorganic	2.28/−2.0	1.4 × 10^7^	500	5 × 10^3^	ECM and VCM	10,000	[139]
PEDOT: PSS/ITO/Rb_6_Pb_5_Cl_16_/Al	Inorganic	−1/1.1	10^6^	500	10^4^	VCM	No	[153]
PET/ITO/Cs_3_Bi_2_Br_9_/Al	Inorganic	−0.45/2.2	10^5^	10^3^	10^4^	VCM and SCLC	100	[142]
ITO/ZnO NCs/b-PEI/Cs_1−x_FA_x_PbBr_3_/V2O_5–y_/Al	Mixed organic and inorganic	5.55/−5.5	–	–	–	VCM	No	[152]

Perovskite quantum dot memristors offer compelling features as an alternative to conventional oxide or 2D-material-based devices due to their tunable bandgap, high carrier mobility, and solution processability. However, they remain constrained by environmental sensitivity and poor endurance. Retention in PVK QDs memristors is often compromised compared to oxides, as perovskite materials degrade under humidity, heat, or electrical stress. As shown in Figure 6d–g, Han et al. reported the best-performing PVK QDs-based memristive architecture with a retention of 4 × 10^5^. At the same time, research does not reveal energy consumption and switching speed, although there is an example of quasi-2D perovskite memristor that achieved an astonishingly high ON/OFF ratio of over 10^9^ [160]. Additionally, Poddar et al. reported a memristor involving PVK quantum wires (monocrystalline), which revealed a switching ratio of ~10^7^ with an ultrafast operating speed of ~100 ps [161]. In comparison, Li et al. fabricated devices with TaOx-based materials, which demonstrated an endurance of more than 10^12^ cycles while operating at a speed of 10 ns (see Table 3) [162]. Similarly, Choi et al. reported that SiO_x_-based memristors had an operational speed of around 100 ps and a retention 10^7^ [163]. Reportedly, the 2D materials-based memristor (Pt/h-BN/Ag) has an endurance of 10^7^ cycles with an operational speed of 50 ns [164].

## 7. Conclusions and Outlook

PVK QD-based memristors are emerging as a compelling platform for next-generation memory technologies, driven by the increasing demand for high-density, flexible, and miniaturized computing systems. The exceptional optoelectronic and ionic properties of PVK QDs at the nanoscale, including bandgap tunability, quantum confinement, and high surface reactivity, position them as promising active materials in resistive switching memory devices.

This review has summarized the core aspects of PVK QD-based memristive systems, including their inherited switching behavior from bulk counterparts, synthetic strategies, and mechanistic switching models. While significant advancements have been made, particularly in exploiting PVK QDs for both electrical and optoelectronic memristors (Table 1), key challenges remain. In particular, a comprehensive understanding of the resistive switching mechanism, especially under optical modulation, remains lacking. This is primarily due to the limited number of mechanistic studies reported for QD-based memory structures. To address this, advanced characterization techniques, such as in situ transmission electron microscopy, cross-sectional imaging, and high-resolution spectroscopic mapping, are recommended to monitor the formation of conductive filaments and ionic movement in real time.

From a fabrication standpoint, solvent-mediated QD deposition introduces switching matrix quality variability, directly impacting device uniformity and reliability. Thicker QD layers typically lead to higher switching voltages, whereas excessively thin films result in inconsistent resistive switching behavior. Moreover, using toxic or low-boiling-point solvents (e.g., hexane, toluene poses challenges in environmental safety and film homogeneity due to the “coffee ring” effect. Therefore, exploring greener, high-boiling-point solvents that are compatible with QDs is crucial for enhancing film quality and device performance. Moreover, while the initial findings have been met with optimism, it should be noted that a significant number of studies have not included exhaustive failure analysis, and the discordant assertions concerning predominant switching mechanisms persist. For instance, the interplay between optical stimulation and ion migration in photo-assisted devices remains to be fully elucidated. Addressing these open questions is imperative for translating laboratory prototypes to practical memory applications.

Scalability is another pressing concern. Conventional spin-coating techniques are insufficient for fabricating large-area or high-resolution memory arrays. Alternative strategies, such as spray coating, inkjet printing, and orthogonal photolithography, should be further investigated to support commercial viability. Notably, the ionic nature of PVK QDs makes them vulnerable to damage during lithographic patterning in polar solvents. Thus, non-polar solvent systems must be employed to ensure structural integrity [165,166].

Employing PVK QDs in flexible memory devices epitomizes a promising dimension meriting further exploration. The PVK QDs advantageously have nanoscale dimensions, which amplify their eligibility for fabricating flexible memory devices. Recent advancements regarding PVK QD application in flexible memory devices revealed remarkable bending durability, thus underscoring the necessity of incessant research [137,139]. Another challenge to staying still in the crossbar array applications is sneaking current; the introduction of the selector (S) or diode (D) has been employed in PVK-based 1S1R or 1D1R architectures, which need to be implemented in PVK QDs [167].

Overall, halide PVK QDs are potential contenders as a functional material matrix in memristive architectures, which is attributed to their unique physio-chemical characteristics. However, futuristic applications require collective efforts from the research community to address the associated bottlenecks. This review could be valuable for applying PVK QDs in memristive devices to cutting-edge intelligent technologies.

## Figures and Tables

**Table 1:** Comparison of the key features between VCM, ECM and interfacial switching.

Device Structure	Switching Voltage (V)	Retention (s)	Filament Material	Ref.
Ag/CH_3_NH_3_PbI_3_/Pt	0.13	1.1 × 10^4^	Ag	[112]
Au/CH_3_NH_3_PbI_3_/ITO/PET	0.7	10^4^	V_I_	[113]
Au/CH_3_NH_3_PbI_3_/Au	0.32	1.17 × 10^4^	V_I_	[114]
Au/Ag/(PEA)_2_(MEA)_n−1_Pb_n_I_3n+1_/PEDOT:PSS/FTO	0.9	10^4^	Formation/dissolution of an AgI monolayer	[115]
Au/Ag/PCBM/CH_3_NH_3_PbI_3_/PEDOT:PSS/FTO	0.25, 0.56	10^5^	Ag^+^	[116]
Au/*α*-CsPbI_3_ QDs/PEDOT:PSS/ITO	1.02	4 × 10^4^	V_I_	[117]
Au/CH_3_NH_3_PbCl_X_I_3−X_/FTO	1.0	2.5 × 10^4^	–	[120]
Ag/CH_3_NH_3_PbI_3−x_Cl_x_/FTO	1.4	4 × 10^4^	Ag	[118]
Ag/CH_3_NH_3_PbI_3_/Pt	–	10^5^	V_I_	[119]
Ag/PMMA@CsPbI_3_/FTO	0.31	10^5^	Ag^+^	[121]
Au/CH_3_NH_3_PbBr_3_/ITO	−0.5	10^4^	MA^+^	[30]
Au/CH_3_NH_3_PbI_3−x_Cl_x_/ITO	0.1	4.68 × 10^4^	hole trapping at PVK/Au interface	[122]
Ag/PMMA/CsSnI_3_/Pt	0.13	7 × 10^3^	Ag	[123]

V_I_: Iodide vacancies.

**Table 3:** Comparison of the best-performing different materials-based memristors.

Configuration	Architecture	Retention (s)	Endurance (Cycles)	Switching Speed	Ref.
Oxide-RRAM	Pt/Ta_2_O_5−x_/TaO_2−x_/Pt	–	10^12^	10 ns	[162]
	Pt/SiO_x_:Pt/Ta	10^7^	3 × 10^7^	<100 ps	[163]
PVK QDs-RRAM	PET/ITO/PMMA/CsPbBr_3_ /PMMA/Ag	4 × 10^5^	5 × 10^3^	–	[139]
2D-RRAM	Pt/h-BN/Ag	–	10^7^	50 ns	[164]
